# Generation of a reporter yellow fever virus for high throughput antiviral assays

**DOI:** 10.1016/j.antiviral.2020.104939

**Published:** 2020-11

**Authors:** Ricardo Sanchez-Velazquez, Giuditta de Lorenzo, Rapeepat Tandavanitj, Chayanee Setthapramote, Peter J. Bredenbeek, Leonia Bozzacco, Margaret R. MacDonald, Jordan J. Clark, Charles M. Rice, Arvind H. Patel, Alain Kohl, Margus Varjak

**Affiliations:** a MRC-University of Glasgow, Centre for Virus Research, Glasgow, UK; b Laboratory of Virology and Infectious Disease, The Rockefeller University, New York, NY, USA

**Keywords:** Yellow fever virus, HiBiT, Luciferase, Drug screen, CPER, YFV, yellow fever virus, scFv, single chain variable fragments, PRNT, plaque reduction neutralization test, CPER, circular polymerase extension reaction, NanoBiT, NanoLuc binary technology, LgBiT, large NanoBiT subunit, MPA, Mycophenolic acid, HDVr, hepatitis delta virus ribozyme, pA, SV40 polyadenylation signal, sE, soluble E protein, TB, test bleed

## Abstract

Yellow fever virus (YFV), a member of the *Flaviviridae* family*,* is an arthropod-borne virus that can cause severe disease in humans with a lethality rate of up to 60%. Since 2017, increases in YFV activity in areas of South America and Africa have been described. Although a vaccine is available, named strain 17D (Theiler and Smith, 1937), it is contraindicated for use in the elderly, expectant mothers, immunocompromised people, among others. To this day there is no antiviral treatment against YFV to reduce the severity of viral infection. Here, we used a circular polymerase extension reaction (CPER)-based reverse genetics approach to generate a full-length reporter virus (YFVhb) by introducing a small HiBit tag in the NS1 protein. The reporter virus replicates at a similar rate to the parental YFV in HuH-7 cells. Using YFVhb, we designed a high throughput antiviral screening luciferase-based assay to identify inhibitors that target any step of the viral replication cycle. We validated our assay by using a range of inhibitors including drugs, immune sera and neutralizing single chain variable fragments (scFv). In light of the recent upsurge in YFV and a potential spread of the virus, this assay is a further tool in the development of antiviral therapy against YFV.

## Highlights

• Bacteria-free approach to rescue yellow fever virus.
• Novel tagged yellow fever virus that permits quantifiable assays.
• Usage of the novel tagged virus for screening of antivirals and immune sera.
• Novel antiviral compounds against YFV were identified.

## Graphical abstract

**Figure:**
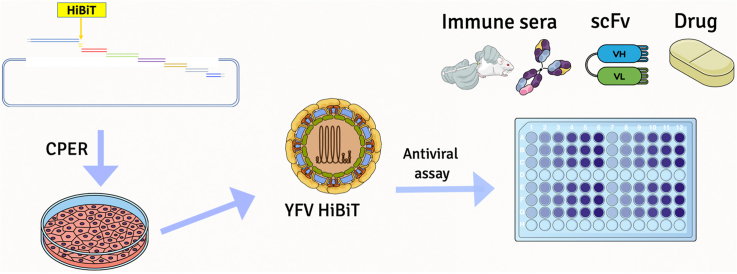

## 1 Introduction

Yellow fever virus (YFV), a *Flaviviridae* family member*,* is an arthropod borne virus mainly transmitted to vertebrate hosts by mosquitoes of the *Aedes* and *Haemagogus* species (Huang, 1986; Monath, 1994; Mutebi and Barrett, 2002; Vasconcelos et al., 2003). In recent years, YFV has remerged in Africa and South America as exemplified by the current worst YFV outbreak in Brazil in years (Goldani, 2017; Jácome et al., 2019; Otshudiema et al., 2017; Possas et al., 2018). Although a vaccine against YFV exists, limitations with the vaccine formulation, severe adverse effects, and shortage of supply have boosted the interest in equally effective yet safer vaccine strategies (Julander et al., 2018; Monath et al., 2010; Pato et al., 2019; Tottey et al., 2018). Moreover, there is no therapeutic treatment to reduce the severity of YFV infections and patients rely on palliative care. Importantly, the lethality of YFV has been reported to be as high as 60% (Monath, 2008; Theiler and Smith, 1937).

Rapid and sensitive assays that are amenable to high-throughput screening are required to identify and assess the efficacy of antiviral therapeutics. The classical way to measure viral neutralization by antibodies is through the plaque reduction neutralization test (PRNT) (Spector and Tauraso, 1968), however, this technique is laborious, time consuming and low-throughput. Replicons of YFV encoding reporter genes have been previously developed (Jones et al., 2005; Queiroz et al., 2013) and used to screen viral inhibitors (Patkar et al., 2009). However, replicons only encode non-structural genes, thus, identification of YFV inhibitors is limited to replication-associated targets, ignoring entry/exit pathways and spread. Importantly, this method cannot screen neutralizing agents (antibodies/antivirals) against the envelope (E) protein, which is the target of most neutralizing antibodies (Vratskikh et al., 2013) and some drugs (Mayhoub et al., 2011). Thus, reporter viruses are better candidates to identify a broader range of inhibitors targeting the virus.

Reporter viruses also have been widely used for the development of high-throughput neutralization assays (Deng et al., 2016; Gadea et al., 2016; Schoggins et al., 2012; Song et al., 2014; Zhang et al., 2016). Nevertheless, the length and size of the most common reporters can interfere with virus replication and result in significant virus attenuation. Although tags have been successfully added in the capsid region, the NS1 gene has been shown to be more tolerable to insertions, which often resulted in a more stable reporter virus (Eyre et al., 2017). An optimal tag should thus minimize dimensional intrusion and provide a quantifiable range of protein expression that is proportional to virus replication. Here we describe the development of a YFV vaccine strain 17D-based reverse genetics system to produce a virus carrying an 11-amino acid-long, HiBiT tag based on *in vitro* bacterium free approach adapted from Setoh et al. (2017). The HiBiT is a component of the NanoLuc binary technology (NanoBiT), but it lacks the enzymatic activity on its own. This activity is restored upon the association of HiBiT with large NanoBiT subunit (LgBiT), which generates an enzyme capable of bioluminescence (Schwinn et al., 2018). The reporter virus (YFVhb) displayed similar growth kinetics and titres in HuH-7 cells to the parental vaccine strain virus. Furthermore, the reporter virus can be used to develop a highly sensitive high-throughput antiviral screening assay (Z values 0.69, signal-to-background ratio >1000). Indeed, we were able identify to new inhibitors of YFV, 7-deaza-2′-C-methyladenosine (7DMA), Celgosivir and Nelfinavir.

## 2 Materials and Methods

### 2.1 Cell lines and virus

BHK-21, HuH-7, Vero, and A549NPro were maintained in Dulbecco's Modified Eagle Medium (Life Technologies) supplemented with 10% fetal bovine serum (FBS) (Life Technologies), 1% non-essential amino acids, penicillin (100 IU/ml) streptomycin (0.1 mg/ml) (Gibco), and 10 mM HEPES buffer (Gibco). Cells were cultured at 37 °C, in 5% CO_2_ under a humidified atmosphere. S2 drosophila cells were maintained in Schneider's complete medium (Gibco) supplemented with 10% FBS, and Penicillin (100 IU/ml) Streptomycin (0.1 mg/ml) at 28 °C. Expi293F cells (Thermo Fisher Scientific) were grown in Expi293™ Expression Medium following manufacturer's instructions. The A549NPro (puromycin resistant) cells expressing IRF3-degrading BVDV NPro were a kind gift from R.E Randall (University of St. Andrews, UK) and maintained in the presence of 2 μg/ml puromycin (Hilton et al., 2006). YFV vaccine strain 17D (catalogue number 0006251v) was acquired from Public Health England (PHE). The parental YFV vaccine strain 17D (YFV-17D) infectious clone used in this study is a derivative of the infectious clone pACNR/FLYF-17Dx (Bredenbeek et al., 2003). The derivative, designated pACNR-2015FLYF-17Da, was created using standard molecular biology techniques and differs from pACNR/FLYF-17Dx by a) the presence of an *Afl*II site for DNA linearization prior to *in vitro* RNA production, b) a C nucleotide (nt) at YFV-17D position 8212, which had originally been mutated to a T (CTCGAG changed to CTTGAG) to allow the use of *Xho*I as the linearization run-off site, and c) a G at nt 4025, rather than an A, since upon comparison of multiple vaccine strain sequences available in Genbank (including deposited sequences for 17DD, 17D-204 and 17D-213), as well as to published 17D-204 deep sequencing data (Beck et al., 2014) this is uniformly a G. Plasmid pACNR-2015FLYF-17Da was verified by complete sequencing and the sequence has been deposited to Genbank (accession number: MT114401).

### 2.2 Reagents

Mycophenolic acid (MPA) was purchased commercially (Merck), and dissolved in methanol or DMSO. 7DMA (Biosynth carbosynth), Sinefungin (Abcam), Ribavirin (Merck), Ivermectin (Merck), ARDP0006 (Merck), Celgosivir (Merck), and Nelfinavir (Merck) were dissolved in DMSO. Pan-flavivirus monoclonal antibody 4G2 (Henchal et al., 1982) was purified from a hybridoma line D1-4G2-4-15 obtained from ATCC (HB-112). Donkey anti-mouse IgG (H+L) Alexa Fluor 488 (A-21202) and Invitrogen-V5 tag Monoclonal antibody (R960-25) were acquired from Thermo Fisher Scientific and goat anti-mouse IgG peroxidase antibody (A4416) from Merck. IRDye 680RD Donkey anti-mouse IgG secondary antibody (926–68072) was obtained from LI-COR Biosciences.

### 2.3 Generation of recombinant reporter virus by CPER

The reporter virus was generated by an adapted CPER method as described previously for ZIKV (Setoh et al., 2017). Seven overlapping fragments covering the entire YFV genome were amplified with the respective primers (Supplementary Table S1) and KOD DNA polymerase (Merck) from YFV-17D infectious clone pACNR-2015FLYF-17Da (Supplementary Sequence S1). An additional overlapping nucleotide fragment containing the HiBiT tag was synthesised and amplified (Supplementary Sequence S2). The overlapping fragments plus the linker fragment, which contains a CMV-promoter, a hepatitis delta virus ribozyme (HDVr), and an SV40 polyadenylation signal (pA) were mixed in equimolar amounts (0.1 pmol) and subjected to CPER with Phusion DNA polymerase (2 min of denaturation at 98 °C; 20 cycles of 10 s at 98 °C, 15 s at 64 °C, and 12 min at 72 °C; and a final extension for 12 min at 72 °C) to circularise the DNA. The crude CPER product mixture was directly transfected into BHK-21 cells using Lipofectamine 2000 (Thermo Fisher Scientific). Infection was allowed to progress for a week before harvesting the media. To amplify the virus, HuH-7 were infected at a multiplicity of infection (MOI) 0.01 for 72 h.

### 2.4 Infection with YFV-17D or YFVhb

Different mammalian cell lines were infected with either YFVhb or YFV-17D (from PHE) at a MOI 0.01 for 1 h. After virus absorption, the inoculum was removed; cells were washed with PBS and incubated for 72–96 h with DMEM supplemented with 2% FBS, 1% non-essential amino acids, penicillin (100 IU/ml) streptomycin (0.1 mg/ml), 10 μM HEPES buffer. Then, virus in the cell supernatant was titrated in a 50% tissue culture infective dose (TCID_50_) assay. For replication kinetics assays with YFVhb, Vero, BHK-21, HuH-7, and A549NPro were seeded at a density of 2 × 10^4^ cells per well in a 96-well plate format overnight. The next day, plates were placed on ice for 30 min before infecting the cells with YFVhb at a MOI of 1 for 1 h on ice to synchronise the infection. Plates were then washed with PBS to remove any unbound virus, and 100 μl of DMEM (2% FBS) was added to the cells. Plates were incubated at 37 °C, 5% CO_2_ under a humidified atmosphere and Nano-Glo HiBiT Lytic Detection System (Promega) was used to measure luminescence on GloMax microplate reader (Promega).

### 2.5 Titration of virus by TCID_50_

HuH-7 cells were infected with 10-fold serial dilutions in heptuplicate in a 96 well plate for 72 h at 37 °C in 5% CO_2_. After incubation, the supernatant was discarded, and cells were fixed in 100% methanol at −20 °C for 2 h. Plates were washed with PBS-0.05% Tween-20 and fixed cells were incubated with pan-flavivirus MAb 4G2 at 3 μg/ml for 1 h, which binds to the virus envelope (E) protein (Henchal et al., 1982). Bound antibodies were detected using goat anti-mouse IgG (H+L)-Alexa Fluor 488 at a dilution of 1:500. The TCID_50_ was calculated using the Spearman-Kärber algorithm (Hierholzer and Killington, 1996).

### 2.6 YFVhb inhibitor and antiviral assay

HuH-7 cells were seeded at a density of 2 × 10^4^ cells/well in a 96-well plate. The following day, cells were infected with YFVhb at MOI 0.01. To test drugs against YFVhb or YFV, cells were pre-incubated with the drug for 1 h prior to infection with the virus and the drug was present during the 48 h course of the experiment. For antibody-mediated neutralization, 4 × 10^2^ YFVhb infectious units were pre-treated with serial dilutions of scFv-5A or mouse sera for 1 h at 37 °C and then cells were incubated with the mixture for 48 h scFv-5A (Genebank accession number AY661701.1) was expressed in drosophila S2 cells and purified as described by Backovic et al. (2010) and Backovic and Krey (2016). Nanoluciferase signal was detected using Nano-Glo HiBiT Lytic Detection System (Promega) utilising a GloMax microplate reader. Cytotoxicity of the panel of drugs was assessed by incubation of the cells with drug under screening conditions, only without virus. Cell proliferation reagent WST-1 (Roche) was used to measure cell viability at different drug concentrations. The data was normalised to the values obtained from incubation with the drug vehicle, DMSO.

### 2.7 Z-factor

For Z-factor determination, 40 replicates of a positive control (MPA 10 μM) and 40 replicates of a negative control (MPA vehicle, methanol) were compared using the YFVhb assay described in this study. Briefly, HuH-7 cells were pre-incubated for 1 h with either 10 μM of MPA or compound vehicle (methanol) at appropriate dilutions. Then, without removing the compound or methanol, YFVhb was added at a MOI 0.01 and incubated for 48 h. The Z-factor was calculated according to the formula previously proposed by Zhang et al. (1999).

### 2.8 Generation of YFV soluble E and immunisation for sera generation

A mammalian codon optimised YFV soluble E protein (sE) lacking the stem region and transmembrane domain (amino acids 394–493) was synthesised. The construct was linked to a N-terminal immunoglobulin leader sequence, a C-terminal V5 tag (GKPIPNPLLGLD) and Strep tag II. The construct was cloned into a pVAX (a kind gift of Dr. Oscar Burrone) vector and transfected using ExpiFectamine™ 293 Transfection kit (Thermo Fisher Scientific). The protein was purified with Streptrap HP column (GE Healthcare) using the ÄKTA Pure (GE Healthcare) system. The concentration of sE protein was determined using NanoDropOne (ThermoScientific). To generate anti-YFV sera, Balb/c mice (n = 6) were subcutaneously immunised with 5 μg of purified YFV sE protein in 60 μl together with 73 μl of aluminium hydroxide-monophosphoryl lipid A as an adjuvant. Mice received two identical boosts of YFV sE at 14 days intervals. Test bleeds were recovered from mice 2 weeks after each boost. Binding of antibodies to YFV E were determined by incubation of serial dilutions of mouse sera to biotinylated YFV E protein in an ELISA format. A goat anti-mouse IgG-peroxidase (Merck) was used as a secondary antibody and TMB as a substrate (Life Technologies).

### 2.9 Western blot protocol

Eluted fractions obtained from purification of the sE protein were separated by 10% SDS-PAGE under reducing conditions for 1.5 h at 100V. The proteins were transferred to a PVDF membrane by a semi-dry transfer 24V for 30 min. The PVDF membrane was blocked using Odyssey Blocking Buffer PBS overnight. As a primary antibody, Invitrogen-V5 tag Monoclonal antibody was added at a dilution of 1:1000 for 1–2 h in blocking buffer. The membrane was washed thrice with PBST before addition of the secondary IRDye 680RD Donkey anti-mouse IgG secondary antibody (LI-COR). The membrane was washed again and digitally developed with LI-COR Image Studio.

### 2.10 Plaque assay-based neutralization assay (PRNT)

Vero cells were seeded at a density of 4 × 10^5^ cells per well in a 12 well plate. The following day, the cells were infected with 80 YFV-17D infectious units previously incubated for 1 h with serial dilutions of mouse sera. Infection was allowed for two hours in 2× DMEM 4% FBS before the addition of overlay medium to reach a final concentration of 3% carboxymethyl cellulose and 1× DMEM. The cells were then incubated for 6–7 days. To develop the plate, cells were gently washed with PBS and fixed with 3.7% formaldehyde in PBS for 10 min, followed by staining with 0.1% crystal violet in 20% ethanol.

## 3 Results

### 3.1 Construction of recombinant reporter virus

**Fig. 1:**
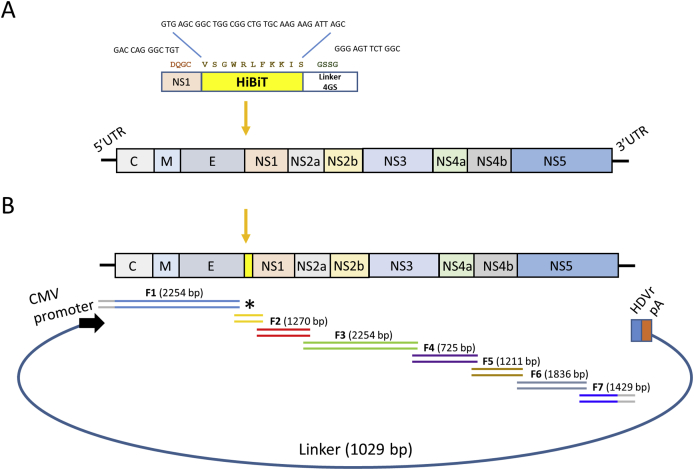
**Addition of a small tag to YFV NS1. (A)** An eleven amino acid reporter tag was placed at the N terminus of the NS1 protein. The reporter fragment consisted of the initial four amino acids of NS1 (DQGC) encoded by different codons to the original sequence, followed by the HiBiT tag (VSGWRLFKKIS), and a 4 GS linker (GSSG). **(B)** The YFV genomic clone was used to amplify 7 fragments, F1–F7, an additional fragment containing the HiBiT reporter tag (*), was also included. These 8 overlapping fragments were mixed in equimolar concentrations with a linker containing a CMV promoter at the 5′ end, a HDVr, and a pA at the 3′ end. The fragments were subjected to CPER to generate circular genomes, which were transfected to BHK-21 cells to produce the reporter virus.

The reporter virus was constructed by the introduction of the small 11-amino acid Nano-Glo HiBiT tag (Schwinn et al., 2018) at the beginning of the non-structural protein 1 gene (NS1), one of the areas shown to best tolerate insertions (Bonaldo et al., 2007; Eyre et al., 2017; Kassar et al., 2017; Tamura et al., 2017). The HiBiT tag was flanked in the N terminus by the first four amino acids of NS1, although encoded by a different codon from the original NS1 to maintain the cleavage site and to avoid recombination that could excise the tag sequence during viral replication (Fig. 1A). Additionally, at the end of the tag, a protein linker GSSG (4 GS) was added to improve solubility and flexibility (Chen et al., 2013b).

To generate the reporter virus YFVhb, we followed the reverse genetics system designed previously (Edmonds et al., 2012; Setoh et al., 2017) named CPER. As shown in Fig. 1B, seven overlapping fragments were amplified from YFV-17D infectious clone pACNR-2015FLYF-17Da. The fragment containing the tag (* in Fig. 1B) was amplified from a synthetic DNA fragment. Altogether these fragments covered the entire reporter virus genome. An additional fragment that contained the CMV promoter (for transcription of RNA), HDVr (to ensure correct 3’ terminal cleavage of RNA), and SV40 pA was included as well (Fig. 1B); these fragments were mixed in equimolar amounts and were subjected to CPER. The CPER products were then transfected into BHK-21 cells and the virus produced (YFVhb) was subjected to a further round of infection in HuH-7 cells to generate a working stock.

### 3.2 Replication kinetics of YFVhb

**Fig. 2:**
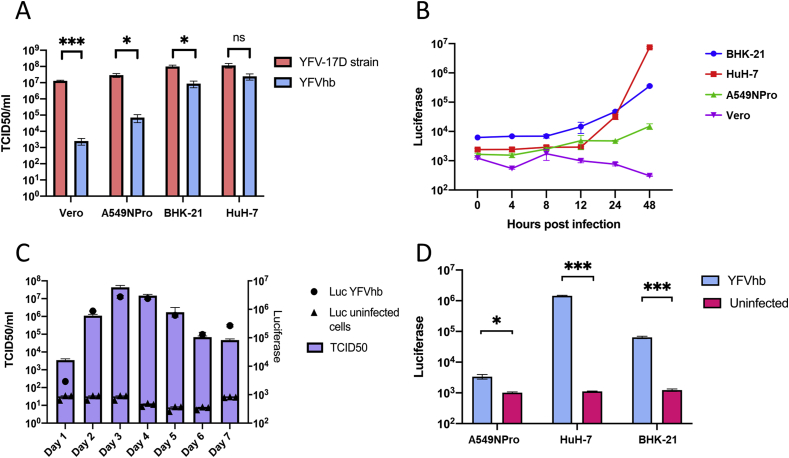
**Comparative growth of YFVhb and YFV-17D. (A)** The indicated cell lines were infected with either YFV-17D or YFVhb at MOI 0.01, at 72 h post infection, supernatant containing virus progeny was titrated by TCID_50_; error bars depict the standard error of the mean (SEM) of three independent titrations**. (B)** Vero, BHK-21, HuH-7, and A549NPro were infected with YFVhb at a MOI of 1 to investigate the replication kinetics of YFVhb. Viral replication was assessed at different time points by measuring the luminescence. Error bars represent SEM from three independent experiments performed in duplicate. **(C)** HuH-7 cells infected with YFVhb at a MOI of 0.01 were monitored daily for TCID_50_ and luciferase (Luc) signal; error bars depict the SEM of the luciferase activity of three independent experiments conducted in triplicate; titration of virus was done in singlicate in three independent experiments. **(D)** Cells were infected with YFVhb at MOI 0.01 or mock-infected with PBS. After 72 h, the luciferase activity was measured. Error bars depict the SEM of the luciferase activity of three independent experiments performed in triplicate.

To compare the growth of YFVhb with the parent vaccine strain YFV-17D, different cell lines were infected at a MOI of 0.01 for 72 h. After incubation, the culture medium was collected, and viral titres were determined by TCID_50_. YFVhb was produced at a considerably lower titre (3–4 logs) in Vero and A549NPro cells than the original YFV-17D strain; A549NPro cells are IRF3 deficient, which can enhance the infectivity and replication of various viruses (Hilton et al., 2006). It was unexpected to see low titres in Vero and A549NPro cells, where parental YFV-17D can efficiently replicate. In HuH-7 cells both the parental and the reporter virus replication reached the same titres after 72 h. In BHK-21, although replication of YFVhb was slightly reduced, the titres were within about 1 log lower of that of YFV-17D (Fig. 2A). To further assess YFVhb replication kinetics, different cell lines were infected with the virus at a higher MOI, the accumulation of HiBiT-tagged NS1 was monitored over the period of 48 h (Fig. 2B). Luminescence levels did not increase over time in Vero cells, there was a slow increase in case of A549NPro cells, which correlates well with the data shown in Fig. 2A. However, the luciferase signal increased steadily in BHK-21 and HuH-7 cells, further suggesting that the presence of the tag on NS1 may have cell type specific effects.

To further correlate YFVhb titres with luciferase signal, HuH-7 cells were infected at a MOI of 0.01, and the TCID_50_ of the released progeny and luciferase were measured over a period of seven days. As shown in Fig. 2C, luciferase signal was found to be proportional to the viral TCID_50_ titres, reaching high activities in second day of infection and staying till day 5. The highest viral titre was reached at 3 days post-infection, however a luciferase signal-to-background ratio of over 1000 was observed as early as day 2 post-infection compared to uninfected cells. To design a sensitive YFV antiviral assay, cell lines producing the best signal-to-background ratio were identified by comparing luminescence between infected and uninfected cell lineages (Fig. 2D). Although both HuH-7 and BHK-21 produced a significant signal-to-background ratio of over 1300 and 50, respectively, HuH-7 provides a wider range of luciferase signal. In addition, the signal-to-noise ratio was worst in the case of A549NPro cells.

### 3.3 Design of an antiviral screening assay using YFVhb

**Fig. 3:**
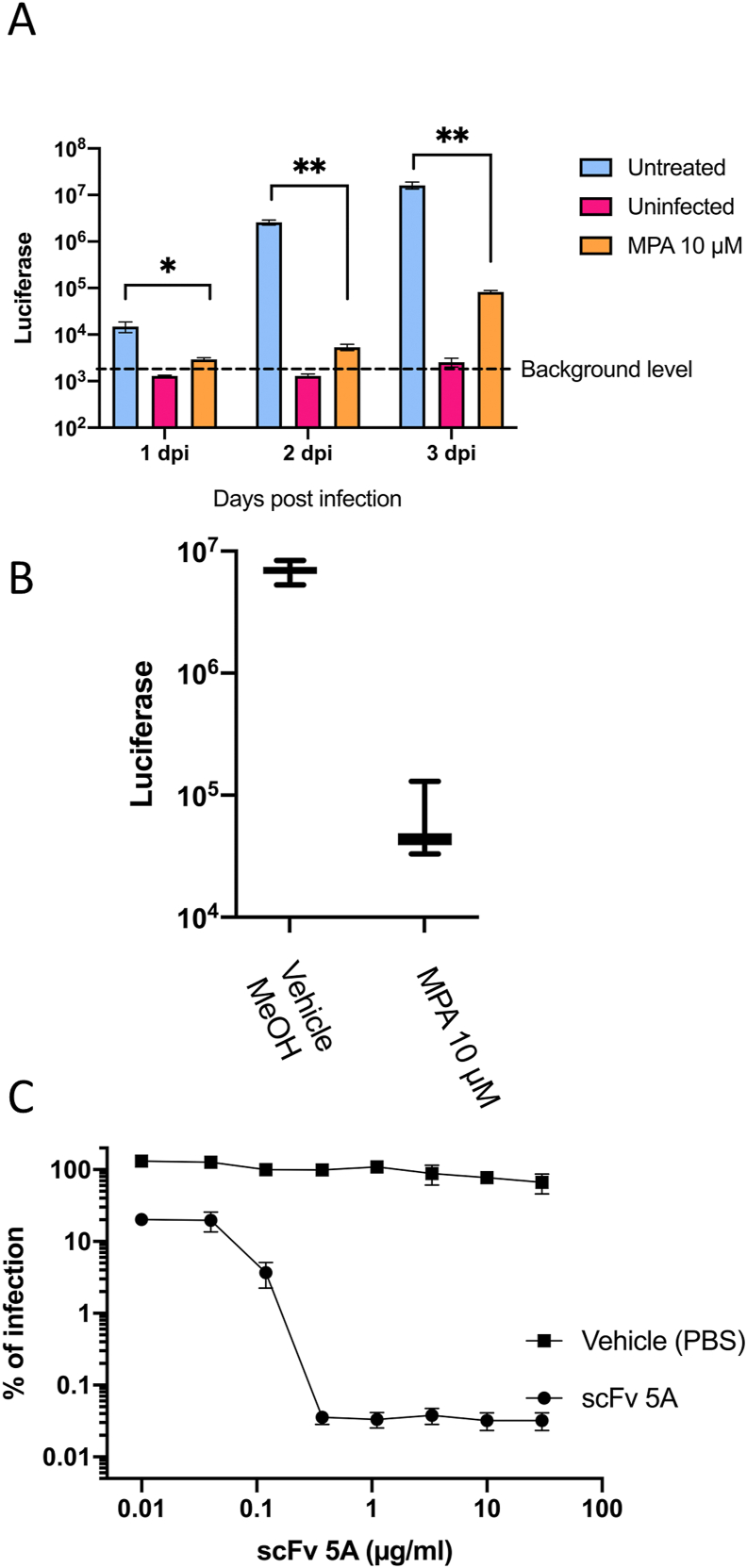
**Testing suitability of YFVhb for screening of antiviral compounds. (A)** HuH-7 cells were either pre-treated with MPA at 10 μM or left untreated for 1 h, cells were then washed and infected with YFVhb, as a negative control, a group of cells were left uninfected to monitor background signal caused by uninfected cells; luciferase activity was measured daily over a period of three days post infection, error bars depict the SEM of the luciferase signal from three independent experiments in duplicate. Statistical significance was determined using T-test without correction for multiple comparisons, with alpha = 0.05, without assuming a consistent SD using GraphPad Prism (**P ≤ 0.01; ***P ≤ 0.001). **(B)** To calculate the Z-factor of the assay, 40 replicates were either treated with 10 μM MPA or MPA vehicle (methanol) and infected with YFVhb. The established parameters of the assay were used, and luciferase was measured 48 h post-infection. **(C)** 400 virus infectious units were incubated with 10-fold serial dilutions of the scFv-5A. Then, the mixture of virus with scFv-5A was used to infect HuH-7 cells for 48 h. A group of cells were treated with an equal volume of scFv vehicle, PBS, before infection and luciferase values were set as 100% infection. Error bars represent SEM of three independent experiments conducted in triplicate.

To identify the optimal length of infection for the antiviral assay, HuH-7 cells were pre-treated with 10 μM MPA, a non-nucleoside inhibitor of inosine monophosphate dehydrogenase that has been shown to inhibit a variety of flaviviruses, including YFV (Barrows et al., 2016; Diamond et al., 2002; Neyts et al., 1996; Sebastian et al., 2011). The IC_50_ value of MPA has been measured to be 0.25 μM by Neyts et al. (1996) in Vero cells, and 0.31 μM by Diamond et al. (2002) in human Hep3B liver cells, respectively. After treatment with MPA, cells were washed with PBS, infected with YFVhb, and the respective luciferase activity was measured over time. As shown in Fig. 3A, at all timepoints, a significantly higher luciferase activity in untreated cells compared to treated cells was observed, showing the sensitivity range of the assay and indicating the suitability of YFVhb for use in quick drug screening assays. Although cells were exposed to an inhibitory MPA concentration, YFV is not fully inhibited unless exposed to the drug continuously as previously reported (Diamond et al., 2002). Consequently, an increase in the luciferase signal at day 3 in MPA-treated cells was observed (Fig. 3A).

### 3.4 Statistical evaluation of the high throughput assay

The dynamic range and data variation obtained from the assay was used to statistically evaluate the suitability of the assay as a high-throughput assay by calculating the Z-factor (Zhang et al., 1999). In addition, the Z-factor may also represent an assay validation by measuring the effectiveness of the assay in the differentiation of two variables, in this case control-infected and drug-treated infection. Overall, the mean luciferase measured in treated cells was >100 times lower than untreated cells (Fig. 3B). A Z-factor of 0.69 was calculated, which categorises this as an excellent high-throughput screening assay according to Zhang et al. (1999).

### 3.5 Utility of the assay for screening of neutralizing antibodies and derivatives

In addition to rapid drug screening, this assay may be used to characterise neutralizing antibodies and their derivatives, such as scFvs. Recently, a potently neutralizing antibody against YFV, named 5A, generated by Daffis et al. (2005), was further characterised by Lu et al. (2019). Here we generated the single-chain variant fragment scFv-5A to test its neutralization activity in our assay; luciferase activities were determined 48 h post-infection. As shown in Fig. 3C, scFv-5A was able to neutralize reporter virus infection in a dose-dependent fashion, exhibiting a 90% inhibitory concentration (IC_90_) value of 0.09 μg/ml. These results are in keeping with those reported previously by Lu et al. (2019).

### 3.6 Screening for anti-YFV compounds

**Fig. 4:**
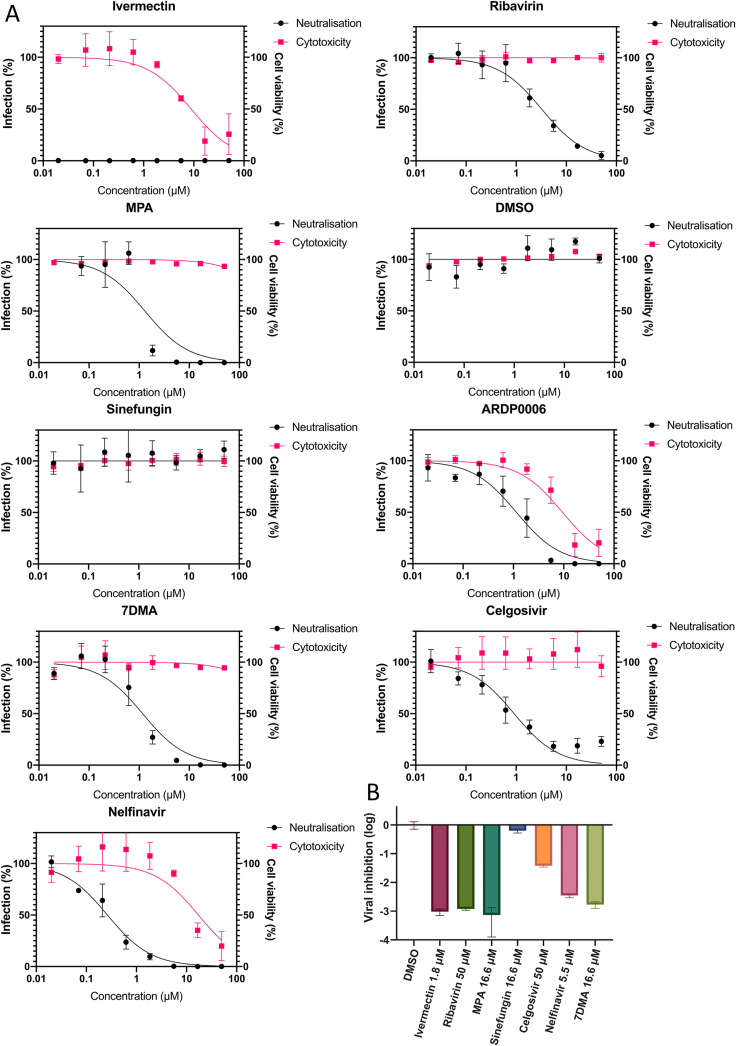
**Inhibition of YFVhb by a panel of drugs. (A)** HuH-7 cells were pre-treated with serial three-fold dilutions of a panel of drugs or their vehicle (DMSO). Cells were infected with YFVhb as described in Materials and Methods. Luciferase readings were normalised to vehicle values, set to 100%. Cell viability was measured with WST-1 reagent (Roche) in a colorimetric assay, and the readings were normalised to vehicle values, set at 100% cell viability. Error bars represent SEM of triplicates in three independent experiments. **(B)** The inhibitory activity of the panel of drugs was tested using the parental YFV-17D by measuring the reduction of viral titres compared to the drug vehicle. Error bars represent the SEM of three independent biological experiments.

Since MPA is a well-known inhibitor of YFV, we aimed to utilize our reporter virus to identify novel antiviral compounds. There are several drugs that are known to inhibit other flaviviruses, and to the best of our knowledge have not been yet tested with YFV. ARDP0006 (protease inhibitor) has been shown to be antiviral against DENV (Tomlinson et al., 2009); another protease inhibitor, Nelfinavir, show activity against JEV, ZIKV and WNV (Wang et al., 2017). Sinefungin, a cap-synthesis inhibitor, can target WNV (Chen et al., 2013a) and DENV (Lim et al., 2015). 7DMA, an adenosine analogue, has been reported to be antiviral against DENV, ZIKV and TBEV (Eyer et al., 2017; Schul et al., 2007; Zmurko et al., 2016). Celgosivir, host alpha-glucosidase inhibitor, helps to misfold DENV NS1 protein (Rathore et al., 2011). NS3 helicase inhibitor, Ivermectin (Mastrangelo et al., 2012), and nucleoside analogues (MPA and Ribavirin) (Diamond et al., 2002; Neyts et al., 1996) were used as positive control, and DMSO as a negative control. HuH-7 cells were treated with different drugs concentrations 1 h pre-infection, and thereafter the cells were infected with YFVhb at a MOI of 0.01; the drug was present throughout the course of experiment, and in parallel a cytotoxicity assay was conducted (Fig. 4A). Of the tested drugs, Sinefungin had no effect on YFVhb, whereas 7DMA and Celgosivir showed low levels of cytotoxicity and inhibition comparable to MPA and Ribavirin. Nelfinavir treatment diminished the luciferase signal, though it was toxic at higher concentrations. ARDP0006 was the most toxic of the compounds and its antiviral effect is more likely due to cell death, thus, it was omitted from further analysis. To confirm that the drugs can target the virus, the most promising compounds were tested with YFV-17D at a single non-toxic concentration. We confirmed 7DMA, Celgosivir, and Nelfinavir as inhibitors of YFV, as measured by the reduced number of released infectious particles from cells (Fig. 4B).

### 3.7 Utility of the assay for screening sera

**Fig. 5:**
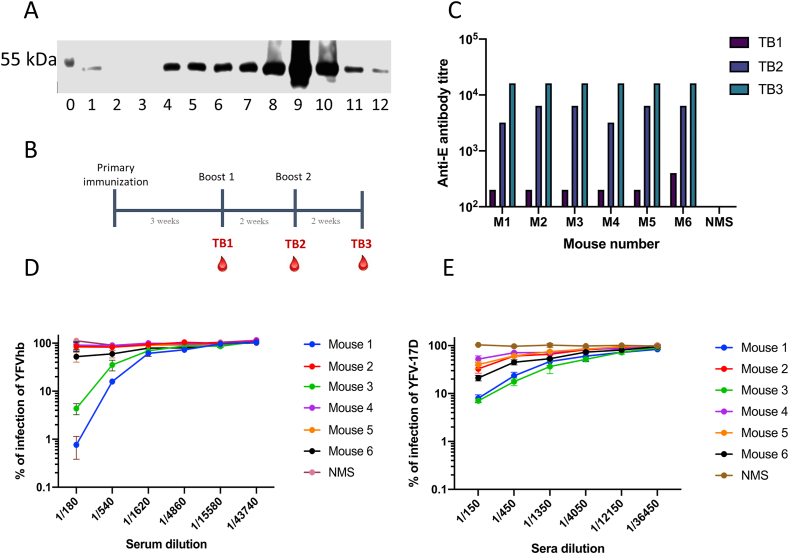
**Inhibition of YFV replication by antibodies from sE-immunised mice. (A)** YFV soluble envelope protein was transfected into Expi293F cells for 5 days. The protein was purified by affinity chromatography. Samples from each step of the purification were separated by SDS-PAGE and analysed by Western blot using a monoclonal anti-V5 tag antibody; 0- molecular weight, 1- cell extract from transfected cells, 2- supernatant from transfected cells, 3- purification flow through, 4–12 fractions eluted from ÄKTA Pure (GE Healthcare) system**. (B)** Mice (n = 6) were immunised with 5 μg of purified YFV soluble E protein. Test bleeds (TB) were taken 2–3 weeks after each immunisation/boost. (**C**) Dilutions of test bleeds obtained throughout the course of the immunisation were tested in an ELISA format for recognition of YFV E. The anti-YFV E titre of each test bleed was defined as the reciprocal of the last dilution which generated a colorimetric signal at least three times higher than normal mouse sera (NMS). **(D)** Dilutions of the sera obtained from mice immunised with YFV sE were mixed with 400 YFVhb infectious units for 1 h before infecting HuH-7 cells. Luciferase activities were determined at 48 h post-infection; error bars represent SEM of three independent experiments done in singlicate. Luciferase produced by NMS was considered 100% infection. **(E)** Dilutions of the sera obtained from mice immunised with sE were mixed with 80 YFV-17D infectious units for 1 h before infecting Vero cells and application of a semi-solid overlay; cells were incubated for 6–7 days and stained with 0.1% crystal violet. The PRNTs were performed three times in singlicate for each serum dilution.

This antiviral test can also be used to determine the generation of protective antibodies in sera from immunised animals. We expressed and purified a subunit soluble envelope (Fig. 5A) for immunisation and administered to six mice. Three test bleeds were carried out and after first and second test bleed the antigen was re-administrated to boost immunisation (Fig. 5B), there was increment of virus E-specific antibody titres in time as measured by ELISA (Fig. 5C), as expected. A curve of inhibition for YFVhb was observed by luciferase measurement; with 2 sera out of 6 showing virus neutralization (Fig. 5D). The neutralization capacity of antibodies from these two animals was further confirmed by PRNT (Fig. 5E) using the parental YFV-17D strain. Overall, our results show that YFVhb can be used in quick antiviral screening assays.

## 4 Discussion

Here, we used reverse genetic system CPER to generate a HiBiT based reporter YFV. The CPER approach has been previously successfully used for ZIKV rescue (Setoh et al., 2017) and tagging with HiBiT has been established for other flaviviruses (Tamura et al., 2017, 2019). Reporter viruses containing a full reporter protein in the capsid region are often unstable, attenuated, or display strong differences in viral growth compared to parental virus (Gadea et al., 2016; Schoggins et al., 2012; Vandergaast et al., 2014; Zou et al., 2011). In comparison, YFVhb showed no difference in replication to parental YFV-17D in HuH-7 and reached comparable titres in BHK-21 cells. Moreover, our data suggest that YFVhb can be used to rapidly screen viral inhibitors and may also be used for the study of replication, dissemination and potentially tissue tropism in animal models as shown by Tamura et al. (2019) for other flaviviruses. We were able to identify three novel inhibitors of YFV (7DMA, Celgosivir and Nelfinavir) using our newly developed reporter virus. These findings were validated by measuring inhibition of the viral titre in parental strain, YFV-17D.

The current techniques to measure YFV inhibition are often low-throughput, unstable, or cannot screen inhibitors against all viral cycle stages. YFVhb described here can be used in rapid drug screening and antibody neutralization assays, as demonstrated in this study. With all inhibitory compounds, the reporter virus displayed a concentration dependent curve of inhibition, which shows the range and robustness of the assay. The inhibitory concentrations of MPA, scFv-5A and YFV sE immunised mice here reported were comparable to results obtained by other classical techniques, which are not amenable for high-throughput assays. Altogether, this assay developed can be a useful tool in the screening of antiviral reagents considerably faster than classical techniques.

In the light of recent major outbreaks in South America, combined with the risk of importing the virus into non-endemic areas, YFV has again become a major public health concern again (Couto-Lima et al., 2017; Faria et al., 2018; Figueiredo, 2019; Jácome et al., 2019; Possas et al., 2018; Vasconcelos and Monath, 2016; Wilder-Smith, 2019). Although susceptible urbanised areas were identified by the WHO for potential YFV outbreaks (E Brent et al., 2018), it is largely envisioned that the current vaccine supply would not provide full coverage. There is an urgent need to develop treatments against YFV infection. Tools and assays developed over the course of this study can support these efforts. A virus with minimum tag or reporter-mediated attenuation should greatly facilitate obtaining meaningful data with antivirals or antibodies/antibody derivatives. The generation of recombinant flaviviruses involves the use of full-length virus cDNA clones, which can be toxic to bacteria during their propagation due to leaky expression from cryptic prokaryotic promoters, resulting in poor yields (Pu et al., 2011). We would envisage that CPER method can be used to insert small tags rapidly into genomes of different (flavi)viruses in multiple locations, which permits a faster response in the emergence of epidemics caused by unknown RNA viruses to screen for potential antiviral candidates.

## 5 Animal work ethical approval

All animal research described in this study was approved by the University of Glasgow Animal Welfare and Ethical Board and was carried out under United Kingdom Home Office Licenses, P9722FD8E, in accordance with the approved guidelines and under the UK Home Office Animals (Scientific Procedures) Act 1986 (ASPA).

## Author contribution

RSV: Data curation, formal analysis, investigation, methodology, validation, visualization, writing-original draft, writing-review and editing. GDL: Conceptualization, methodology. RT: Methodology, formal analysis. CS: Validation. PJB: Investigation, methodology. LB: Investigation, methodology. MRM: Investigation, methodology. JJC: Resources, methodology. CMR: Conceptualization, resources, supervision. AP: Funding acquisition, conceptualization, resources, project administrator, writing-review and editing. AK: Funding acquisition, conceptualization, resources, writing-review and editing. MV: Conceptualization, methodology, supervision, writing-original draft, writing-review and editing.
