# Supplementary material for: Generation of a reporter yellow fever virus for high throughput antiviral assays
Source: Antiviral Res. 2020 Nov;183:104939. doi: 10.1016/j.antiviral.2020.104939 (PMC7649875; doi:10.1016/j.antiviral.2020.104939)
Supplement: Multimedia component 1 [file mmc1.docx]

**Supplementary Table S1.** Primers used to amplify YFV genome overlapping fragments for CPER.

| **Fragment** | **Sequence (5’ TO 3’)** | **Tm °C** | **GC %** | **Length (bases)** |
| --- | --- | --- | --- | --- |
| **F1F**  **F1R** | AGTAAATCCTGTGTGCTAATTGAGGTG  GAAGAACCCTCCAGCGGAGCTG | 56.7  60.4 | 40.7  63.6 | 27  22 |
| **F*F**  **F*R** | CAGCTCCGCTGGAGGGTTCTTC  CCACACTTCCCTTCTTCAAAAGAGGC | 60.4  59.5 | 63.6  50 | 22  26 |
| **F2F**  **F2R** | GCCTCTTTTGAAGAAGGGAAGTGTGG  TCACGCCACCCAAGGCAATCTC | 59.5  58.6 | 50  59.1 | 26  22 |
| **F3F**  **F3R** | GAGATTGCCTTGGGTGGCGTGA  CTGTTCAGGACCACCACACTC | 58.6  56.3 | 59.1  57.1 | 22  21 |
| **F4F**  **F4R** | GAGTGTGGTGGTCCTGAACAG  CAGCAGCTCCCCTCCTACC | 56.3  57.6 | 59.1  68.4 | 22  19 |
| **F5F**  **F5R** | GGTAGGAGGGGAGCTGCTG  GCTCCCCCGGCGTCCAGTTTT | 57.6  60.2 | 68.4  66.7 | 19  21 |
| **F6F**  **F6R** | AAAACTGGACGCCGGGGGAGC  CACTACCTGCCCGGATCCTCTCTGG | 60.2  64.2 | 66.7  64 | 21  25 |
| **F7F**  **F7R** | CCAGAGAGGATCCGGGCAGGTAGTG  AGTGGTTTTGTGTTTGTCATCCAAAGGTCTGC | 64.2  61.8 | 62  43.8 | 25  32 |
| **LinkF** | GCAGACCTTTGGATGACAAACACAAAACCACTGGGTCGGCATGGCATC | 72.2 | 52.1 | 48 |
| **LinkR** | CACCTCAATTAGCACACAGGATTTACTCGGTTCACTAAACGAGCTCTGCTTATATAGACCTCCC | 73 | 45 | 64 |
