# Supplementary material for: Generation of a reporter yellow fever virus for high throughput antiviral assays
Source: Antiviral Res. 2020 Nov;183:104939. doi: 10.1016/j.antiviral.2020.104939 (PMC7649875; doi:10.1016/j.antiviral.2020.104939)
Supplement: Multimedia component 2 [file mmc2.docx]

**Supplementary Sequence S1. Binding sites of primers in plasmid pACNR-2015FLYF-17Da.** The primer binding sites are show in **bold** typeface.

**AGTAAATCCTGTGTGCTAATTGAGGTG**CATTGGTCTGCAAATCGAGTTGCTAGGCAATAAACACATTTGGATTAATTTTAATCGTTCGTTGAGCGATTAGCAGAGAACTGACCAGAACATGTCTGGTCGTAAAGCTCAGGGAAAAACCCTGGGCGTCAATATGGTACGACGAGGAGTTCGCTCCTTGTCAAACAAAATAAAACAAAAAACAAAACAAATTGGAAACAGACCTGGACCTTCAAGAGGTGTTCAAGGATTTATCTTTTTCTTTTTGTTCAACATTTTGACTGGAAAAAAGATCACAGCCCACCTAAAGAGGTTGTGGAAAATGCTGGACCCAAGACAAGGCTTGGCTGTTCTAAGGAAAGTCAAGAGAGTGGTGGCCAGTTTGATGAGAGGATTGTCCTCAAGGAAACGCCGTTCCCATGATGTTCTGACTGTGCAATTCCTAATTTTGGGAATGCTGTTGATGACGGGTGGAGTGACCTTGGTGCGGAAAAACAGATGGTTGCTCCTAAATGTGACATCTGAGGACCTCGGGAAAACATTCTCTGTGGGCACAGGCAACTGCACAACAAACATTTTGGAAGCCAAGTACTGGTGCCCAGACTCAATGGAATACAACTGTCCCAATCTCAGTCCAAGAGAGGAGCCAGATGACATTGATTGCTGGTGCTATGGGGTGGAAAACGTTAGAGTCGCATATGGTAAGTGTGACTCAGCAGGCAGGTCTAGGAGGTCAAGAAGGGCCATTGACTTGCCTACGCATGAAAACCATGGTTTGAAGACCCGGCAAGAAAAATGGATGACTGGAAGAATGGGTGAAAGGCAACTCCAAAAGATTGAGAGATGGTTCGTGAGGAACCCCTTTTTTGCAGTGACGGCTCTGACCATTGCCTACCTTGTGGGAAGCAACATGACGCAACGAGTCGTGATTGCCCTACTGGTCTTGGCTGTTGGTCCGGCCTACTCAGCTCACTGCATTGGAATTACTGACAGGGATTTCATTGAGGGGGTGCATGGAGGAACTTGGGTTTCAGCTACCCTGGAGCAAGACAAGTGTGTCACTGTTATGGCCCCTGACAAGCCTTCATTGGACATCTCACTAGAGACAGTAGCCATTGATAGACCTGCTGAGGTGAGGAAAGTGTGTTACAATGCAGTTCTCACTCATGTGAAGATTAATGACAAGTGCCCCAGCACTGGAGAGGCCCACCTAGCTGAAGAGAACGAAGGGGACAATGCGTGCAAGCGCACTTATTCTGATAGAGGCTGGGGCAATGGCTGTGGCCTATTTGGGAAAGGGAGCATTGTGGCATGCGCCAAATTCACTTGTGCCAAATCCATGAGTTTGTTTGAGGTTGATCAGACCAAAATTCAGTATGTCATCAGAGCACAATTGCATGTAGGGGCCAAGCAGGAAAATTGGAATACCGACATTAAGACTCTCAAGTTTGATGCCCTGTCAGGCTCCCAGGAAGTCGAGTTCATTGGGTATGGAAAAGCTACACTGGAATGCCAGGTGCAAACTGCGGTGGACTTTGGTAACAGTTACATCGCTGAGATGGAAACAGAGAGCTGGATAGTGGACAGACAGTGGGCCCAGGACTTGACCCTGCCATGGCAGAGTGGAAGTGGCGGGGTGTGGAGAGAGATGCATCATCTTGTCGAATTTGAACCTCCGCATGCCGCCACTATCAGAGTACTGGCCCTGGGAAACCAGGAAGGCTCCTTGAAAACAGCTCTTACTGGCGCAATGAGGGTTACAAAGGACACAAATGACAACAACCTTTACAAACTACATGGTGGACATGTTTCTTGCAGAGTGAAATTGTCAGCTTTGACACTCAAGGGGACATCCTACAAAATATGCACTGACAAAATGTTTTTTGTCAAGAACCCAACTGACACTGGCCATGGCACTGTTGTGATGCAGGTGAAAGTGTCAAAAGGAGCCCCCTGCAGGATTCCAGTGATAGTAGCTGATGATCTTACAGCGGCAATCAATAAAGGCATTTTGGTTACAGTTAACCCCATCGCCTCAACCAATGATGATGAAGTGCTGATTGAGGTGAACCCACCTTTTGGAGACAGCTACATTATCGTTGGGAGAGGAGATTCACGTCTCACTTACCAGTGGCACAAAGAGGGAAGCTCAATAGGAAAGTTGTTCACTCAGACCATGAAAGGCGTGGAACGCCTGGCCGTCATGGGAGACACCGCCTGGGATTT**CAGCTCCGCTGGAGGGTTCTTC**ACTTCGGTTGGGAAAGGAATTCATACGGTGTTTGGCTCTGCCTTTCAGGGGCTATTTGGCGGCTTGAACTGGATAACAAAGGTCATCATGGGGGCGGTACTTATATGGGTTGGCATCAACACAAGAAACATGACAATGTCCATGAGCATGATCTTGGTAGGAGTGATCATGATGTTTTTGTCTCTAGGAGTTGGGGCGGATCAAGGATGCGCCATCAACTTTGGCAAGAGAGAGCTCAAGTGCGGAGATGGTATCTTCATATTTAGAGACTCTGATGACTGGCTGAACAAGTACTCATACTATCCAGAAGATCCTGTGAAGCTTGCATCAATAGTGAAA**GCCTCTTTTGAAGAAGGGAAGTGTGG**CCTAAATTCAGTTGACTCCCTTGAGCATGAGATGTGGAGAAGCAGGGCAGATGAGATCAATGCCATTTTTGAGGAAAACGAGGTGGACATTTCTGTTGTCGTGCAGGATCCAAAGAATGTTTACCAGAGAGGAACTCATCCATTTTCCAGAATTCGGGATGGTCTGCAGTATGGTTGGAAGACTTGGGGTAAGAACCTTGTGTTCTCCCCAGGGAGGAAGAATGGAAGCTTCATCATAGATGGAAAGTCCAGGAAAGAATGCCCGTTTTCAAACCGGGTCTGGAATTCTTTCCAGATAGAGGAGTTTGGGACGGGAGTGTTCACCACACGCGTGTACATGGACGCAGTCTTTGAATACACCATAGACTGCGATGGATCTATCTTGGGTGCAGCGGTGAACGGAAAAAAGAGTGCCCATGGCTCTCCAACATTTTGGATGGGAAGTCATGAAGTAAATGGGACATGGATGATCCACACCTTGGAGGCATTAGATTACAAGGAGTGTGAGTGGCCACTGACACATACGATTGGAACATCAGTTGAAGAGAGTGAAATGTTCATGCCGAGATCAATCGGAGGCCCAGTTAGCTCTCACAATCATATCCCTGGATACAAGGTTCAGACGAACGGACCTTGGATGCAGGTACCACTAGAAGTGAAGAGAGAAGCTTGCCCAGGGACTAGCGTGATCATTGATGGCAACTGTGATGGACGGGGAAAATCAACCAGATCCACCACGGATAGCGGGAAAGTTATTCCTGAATGGTGTTGCCGCTCCTGCACAATGCCGCCTGTGAGCTTCCATGGTAGTGATGGGTGTTGGTATCCCATGGAAATTAGGCCAAGGAAAACGCATGAAAGCCATCTGGTGCGCTCCTGGGTTACAGCTGGAGAAATACATGCTGTCCCTTTTGGTTTGGTGAGCATGATGATAGCAATGGAAGTGGTCCTAAGGAAAAGACAGGGACCAAAGCAAATGTTGGTTGGAGGAGTAGTGCTCTTGGGAGCAATGCTGGTCGGGCAAGTAACTCTCCTTGATTTGCTGAAACTCACAGTGGCTGTGGGATTGCATTTCCATGAGATGAACAATGGAGGAGACGCCATGTATATGGCGTTGATTGCTGCCTTTTCAATCAGACCAGGGCTGCTCATCGGCTTTGGGCTCAGGACCCTATGGAGCCCTCGGGAACGCCTTGTGCTGACCCTAGGAGCAGCCATGGTG**GAGATTGCCTTGGGTGGCGTGA**TGGGCGGCCTGTGGAAGTATCTAAATGCAGTTTCTCTCTGCATCCTGACAATAAATGCTGTTGCTTCTAGGAAAGCATCAAATACCATCTTGCCCCTCATGGCTCTGTTGACACCTGTCACTATGGCTGAGGTGAGACTTGCCGCAATGTTCTTTTGTGCCGTGGTTATCATAGGGGTCCTTCACCAGAATTTCAAGGACACCTCCATGCAGAAGACTATACCTCTGGTGGCCCTCACACTCACATCTTACCTGGGCTTGACACAACCTTTTTTGGGCCTGTGTGCATTTCTGGCAACCCGCATATTTGGGCGAAGGAGTATCCCAGTGAATGAGGCACTCGCAGCAGCTGGTCTAGTGGGAGTGCTGGCAGGACTGGCTTTTCAGGAGATGGAGAACTTCCTTGGTCCGATTGCAGTTGGAGGACTCCTGATGATGCTGGTTAGCGTGGCTGGGAGGGTGGATGGGCTAGAGCTCAAGAAGCTTGGTGAAGTTTCATGGGAAGAGGAGGCGGAGATCAGCGGGAGTTCCGCCCGCTATGATGTGGCACTCAGTGAACAAGGGGAGTTCAAGCTGCTTTCTGAAGAGAAAGTGCCATGGGACCAGGTTGTGATGACCTCGCTGGCCTTGGTTGGGGCTGCCCTCCATCCATTTGCTCTTCTGCTGGTCCTTGCTGGGTGGCTGTTTCATGTCAGGGGAGCTAGGAGAAGTGGGGATGTCTTGTGGGATATTCCCACTCCTAAGATCATCGAGGAATGTGAACATCTGGAGGATGGGATTTATGGCATATTCCAGTCAACCTTCTTGGGGGCCTCCCAGCGAGGAGTGGGAGTGGCACAGGGAGGGGTGTTCCACACAATGTGGCATGTCACAAGAGGAGCTTTCCTTGTCAGGAATGGCAAGAAGTTGATTCCATCTTGGGCTTCAGTAAAGGAAGACCTTGTCGCCTATGGTGGCTCATGGAAGTTGGAAGGCAGATGGGATGGAGAGGAAGAGGTCCAGTTGATCGCGGCTGTTCCAGGAAAGAACGTGGTCAACGTCCAGACAAAACCGAGCTTGTTCAAAGTGAGGAATGGGGGAGAAATCGGGGCTGTCGCTCTTGACTATCCGAGTGGCACTTCAGGATCTCCTATTGTTAACAGGAACGGAGAGGTGATTGGGCTGTACGGCAATGGCATCCTTGTCGGTGACAACTCCTTCGTGTCCGCCATATCCCAGACTGAGGTGAAGGAAGAAGGAAAGGAGGAGCTCCAAGAGATCCCGACAATGCTAAAGAAAGGAATGACAACTGTCCTTGATTTTCATCCTGGAGCTGGGAAGACAAGACGTTTCCTCCCACAGATCTTGGCCGAGTGCGCACGGAGACGCTTGCGCACTCTTGTGTTGGCCCCCACCAGGGTTGTTCTTTCTGAAATGAAGGAGGCTTTTCACGGCCTGGACGTGAAATTCCACACACAGGCTTTTTCCGCTCACGGCAGCGGGAGAGAAGTCATTGATGCCATGTGCCATGCCACCCTAACTTACAGGATGTTGGAACCAACTAGGGTTGTTAACTGGGAAGTGATCATTATGGATGAAGCCCATTTTTTGGATCCAGCTAGCATAGCCGCTAGAGGTTGGGCAGCGCACAGAGCTAGGGCAAATGAAAGTGCAACAATCTTGATGACAGCCACACCGCCTGGGACTAGTGATGAATTTCCACATTCAAATGGTGAAATAGAAGATGTTCAAACGGACATACCCAGTGAGCCCTGGAACACAGGGCATGACTGGATCCTGGCTGACAAAAGGCCCACGGCATGGTTCCTTCCATCCATCAGAGCTGCAAATGTCATGGCTGCCTCTTTGCGTAAGGCTGGAAA**GAGTGTGGTGGTCCTGAACAG**GAAAACCTTTGAGAGAGAATACCCCACGATAAAGCAGAAGAAACCTGACTTTATATTGGCCACTGACATAGCTGAAATGGGAGCCAACCTTTGCGTGGAGCGAGTGCTGGATTGCAGGACGGCTTTTAAGCCTGTGCTTGTGGATGAAGGGAGGAAGGTGGCAATAAAAGGGCCACTTCGTATCTCCGCATCCTCTGCTGCTCAAAGGAGGGGGCGCATTGGGAGAAATCCCAACAGAGATGGAGACTCATACTACTATTCTGAGCCTACAAGTGAAAATAATGCCCACCACGTCTGCTGGTTGGAGGCCTCAATGCTCTTGGACAACATGGAGGTGAGGGGTGGAATGGTCGCCCCACTCTATGGCGTTGAAGGAACTAAAACACCAGTTTCCCCTGGTGAAATGAGACTGAGGGATGACCAGAGGAAAGTCTTCAGAGAACTAGTGAGGAATTGTGACCTGCCCGTTTGGCTTTCGTGGCAAGTGGCCAAGGCTGGTTTGAAGACGAATGATCGTAAGTGGTGTTTTGAAGGCCCTGAGGAACATGAGATCTTGAATGACAGCGGTGAAACAGTGAAGTGCAGGGCTCCTGGAGGAGCAAAGAAGCCTCTGCGCCCAAGGTGGTGTGATGAAAGGGTGTCATCTGACCAGAGTGCGCTGTCTGAATTTATTAAGTTTGCTGAA**GGTAGGAGGGGAGCTGCTG**AAGTGCTAGTTGTGCTGAGTGAACTCCCTGATTTCCTGGCTAAAAAAGGTGGAGAGGCAATGGATACCATCAGTGTGTTCCTCCACTCTGAGGAAGGCTCTAGGGCTTACCGCAATGCACTATCAATGATGCCTGAGGCAATGACAATAGTCATGCTGTTTATACTGGCTGGACTACTGACATCGGGAATGGTCATCTTTTTCATGTCTCCCAAAGGCATCAGTAGAATGTCTATGGCGATGGGCACAATGGCCGGCTGTGGATATCTCATGTTCCTTGGAGGCGTCAAACCCACTCACATCTCCTATGTCATGCTCATATTCTTTGTCCTGATGGTGGTTGTGATCCCCGAGCCAGGGCAACAAAGGTCCATCCAAGACAACCAAGTGGCATACCTCATTATTGGCATCCTGACGCTGGTTTCAGCGGTGGCAGCCAACGAGCTAGGCATGCTGGAGAAAACCAAAGAGGACCTCTTTGGGAAGAAGAACTTAATTCCATCTAGTGCTTCACCCTGGAGTTGGCCGGATCTTGACCTGAAGCCAGGAGCTGCCTGGACAGTGTACGTTGGCATTGTTACAATGCTCTCTCCAATGTTGCACCACTGGATCAAAGTCGAATATGGCAACCTGTCTCTGTCTGGAATAGCCCAGTCAGCCTCAGTCCTTTCTTTCATGGACAAGGGGATACCATTCATGAAGATGAATATCTCGGTCATAATGCTGCTGGTCAGTGGCTGGAATTCAATAACAGTGATGCCTCTGCTCTGTGGCATAGGGTGCGCCATGCTCCACTGGTCTCTCATTTTACCTGGAATCAAAGCGCAGCAGTCAAAGCTTGCACAGAGAAGGGTGTTCCATGGCGTTGCCAAGAACCCTGTGGTTGATGGGAATCCAACAGTTGACATTGAGGAAGCTCCTGAAATGCCTGCCCTTTATGAGAAGAAACTGGCTCTATATCTCCTTCTTGCTCTCAGCCTAGCTTCTGTTGCCATGTGCAGAACGCCCTTTTCATTGGCTGAAGGCATTGTCCTAGCATCAGCTGCCTTAGGGCCGCTCATAGAGGGAAACACCAGCCTTCTTTGGAATGGACCCATGGCTGTCTCCATGACAGGAGTCATGAGGGGGAATCACTATGCTTTTGTGGGAGTCATGTACAATCTATGGAAGATG**AAAACTGGACGCCGGGGGAGC**GCGAATGGAAAAACTTTGGGTGAAGTCTGGAAGAGGGAACTGAATCTGTTGGACAAGCGACAGTTTGAGTTGTATAAAAGGACCGACATTGTGGAGGTGGATCGTGATACGGCACGCAGGCATTTGGCCGAAGGGAAGGTGGACACCGGGGTGGCGGTCTCCAGGGGGACCGCAAAGTTAAGGTGGTTCCATGAGCGTGGCTATGTCAAGCTGGAAGGTAGGGTGATTGACCTGGGGTGTGGCCGCGGAGGCTGGTGTTACTACGCTGCTGCGCAAAAGGAAGTGAGTGGGGTCAAAGGATTTACTCTTGGAAGAGACGGCCATGAGAAACCCATGAATGTGCAAAGTCTGGGATGGAACATCATCACCTTCAAGGACAAAACTGATATCCACCGCCTAGAACCAGTGAAATGTGACACCCTTTTGTGTGACATTGGAGAGTCATCATCGTCATCGGTCACAGAGGGGGAAAGGACCGTGAGAGTTCTTGATACTGTAGAAAAATGGCTGGCTTGTGGGGTTGACAACTTCTGTGTGAAGGTGTTAGCTCCATACATGCCAGATGTTCTCGAGAAACTGGAATTGCTCCAAAGGAGGTTTGGCGGAACAGTGATCAGGAACCCTCTCTCCAGGAATTCCACTCATGAAATGTACTACGTGTCTGGAGCCCGCAGCAATGTCACATTTACTGTGAACCAAACATCCCGCCTCCTGATGAGGAGAATGAGGCGTCCAACTGGAAAAGTGACCCTGGAGGCTGACGTCATCCTCCCAATTGGGACACGCAGTGTTGAGACAGACAAGGGACCCCTGGACAAAGAGGCCATAGAAGAAAGGGTTGAGAGGATAAAATCTGAGTACATGACCTCTTGGTTTTATGACAATGACAACCCCTACAGGACCTGGCACTACTGTGGCTCCTATGTCACAAAAACCTCAGGAAGTGCGGCGAGCATGGTAAATGGTGTTATTAAAATTCTGACATATCCATGGGACAGGATAGAGGAGGTCACAAGAATGGCAATGACTGACACAACCCCTTTTGGACAGCAAAGAGTGTTTAAAGAAAAAGTTGACACCAGAGCAAAGGATCCACCAGCGGGAACTAGGAAGATCATGAAAGTTGTCAACAGGTGGCTGTTCCGCCACCTGGCCAGAGAAAAGAACCCCAGACTGTGCACAAAGGAAGAATTTATTGCAAAAGTCCGAAGTCATGCAGCCATTGGAGCTTACCTGGAAGAACAAGAACAGTGGAAGACTGCCAATGAGGCTGTCCAAGACCCAAAGTTCTGGGAACTGGTGGATGAAGAAAGGAAGCTGCACCAACAAGGCAGGTGTCGGACTTGTGTGTACAACATGATGGGGAAAAGAGAGAAGAAGCTGTCAGAGTTTGGGAAAGCAAAGGGAAGCCGTGCCATATGGTATATGTGGCTGGGAGCGCGGTATCTTGAGTTTGAGGCCCTGGGATTCCTGAATGAGGACCATTGGGCTTCCAGGGAAAACTCAGGAGGAGGAGTGGAAGGCATTGGCTTACAATACCTAGGATATGTGATCAGAGACCTGGCTGCAATGGATGGTGGTGGATTCTACGCGGATGACACCGCTGGATGGGACACGCGCATCACAGAGGCAGACCTTGATGATGAACAGGAGATCTTGAACTACATGAGCCCACATCACAAAAAACTGGCACAAGCAGTGATGGAAATGACATACAAGAACAAAGTGGTGAAAGTGTTGAGACCAGCCCCAGGAGGGAAAGCCTACATGGATGTCATAAGTCGACGAGA**CCAGAGAGGATCCGGGCAGGTAGTG**ACTTATGCTCTGAACACCATCACCAACTTGAAAGTCCAATTGATCAGAATGGCAGAAGCAGAGATGGTGATACATCACCAACATGTTCAAGATTGTGATGAATCAGTTCTGACCAGGCTGGAGGCATGGCTCACTGAGCACGGATGTGACAGACTGAAGAGGATGGCGGTGAGTGGAGACGACTGTGTGGTCCGGCCCATCGATGACAGGTTCGGCCTGGCCCTGTCCCATCTCAACGCCATGTCCAAGGTTAGAAAGGACATATCTGAATGGCAGCCATCAAAAGGGTGGAATGATTGGGAGAATGTGCCCTTCTGTTCCCACCACTTCCATGAACTACAGCTGAAGGATGGCAGGAGGATTGTGGTGCCTTGCCGAGAACAGGACGAGCTCATTGGGAGAGGAAGGGTGTCTCCAGGAAACGGCTGGATGATCAAGGAAACAGCTTGCCTCAGCAAAGCCTATGCCAACATGTGGTCACTGATGTATTTTCACAAAAGGGACATGAGGCTACTGTCATTGGCTGTTTCCTCAGCTGTTCCCACCTCATGGGTTCCACAAGGACGCACAACATGGTCGATTCATGGGAAAGGGGAGTGGATGACCACGGAAGACATGCTTGAGGTGTGGAACAGAGTATGGATAACCAACAACCCACACATGCAGGACAAGACAATGGTGAAAAAATGGAGAGATGTCCCTTATCTAACCAAGAGACAAGACAAGCTGTGCGGATCACTGATTGGAATGACCAATAGGGCCACCTGGGCCTCCCACATCCATTTAGTCATCCATCGTATCCGAACGCTGATTGGACAGGAGAAATACACTGACTACCTAACAGTCATGGACAGGTATTCTGTGGATGCTGACCTGCAACTGGGTGAGCTTATCTGAAACACCATCTAACAGGAATAACCGGGATACAAACCACGGGTGGAGAACCGGACTCCCCACAACCTGAAACCGGGATATAAACCACGGCTGGAGAACCGGACTCCGCACTTAAAATGAAACAGAAACCGGGATAAAAACTACGGATGGAGAACCGGACTCCACACATTGAGACAGAAGAAGTTGTCAGCCCAGAACCCCACACGAGTTTTGCCACTGCTAAGCTGTGAGGCAGTGCAGGCTGGGACAGCCGACCTCCAGGTTGCGAAAAACCTGGTTTCTGGGACCTCCCACCCCAGAGTAAAAAGAACGGAGCCTCCGCTACCACCCTCCCACGTGGTGGTAGAAAGACGGGGTCTAGAGGTTAGAGGAGACCCTCCAGGGAACAAATAGTGGGACCATATTGACGCCAGGGAAAGACCGGAGTGGTTCTCTGCTTTTCCTCCAGAGGTCTGTGAGCACAGTTTGCTCAAGAATAA**GCAGACCTTTGGATGACAAACACAAAA**CCACTTAAGCTCGAGCAAGACGTTTCCCGTTGAATATGGCTCATAACACCCCTTGTATTACTGTTTATGTAAGCAGACAGTTTTATTGTTCATGATGATATATTTTTATCTTGTGCAATGTAACATCAGAGATTTTGAGACACAACGTGGCTTTGTTGAATAAATCGAACTTTTGCTGAGTTGAAGGATCAGATCACGCATCTTCCCGACAACGCAGACCGTTCCGTGGCAAAGCAAAAGTTCAAAATCACCAACTGGTCCACCTACAACAAAGCTCTCATCAACCGTGGCTCCCTCACTTTCTGGCTGGATGATGGGGCGATTCAGGCCTGGTATGAGTCAGCAACACCTTCTTCACGAGGCAGACCTCAGCGCTAGCGGAGTGTATACTGGCTTACTATGTTGGCACTGATGAGGGTGTCAGTGAAGTGCTTCATGTGGCAGGAGAAAAAAGGCTGCACCGGTGCGTCAGCAGAATATGTGATACAGGATATATTCCGCTTCCTCGCTCACTGACTCGCTACGCTCGGTCGTTCGACTGCGGCGAGCGGAAATGGCTTACGAACGGGGCGGAGATTTCCTGGAAGATGCCAGGAAGATACTTAACAGGGAAGTGAGAGGGCCGCGGCAAAGCCGTTTTTCCATAGGCTCCGCCCCCCTGACAAGCATCACGAAATCTGACGCTCAAATCAGTGGTGGCGAAACCCGACAGGACTATAAAGATACCAGGCGTTTCCCCTGGCGGCTCCCTCGTGCGCTCTCCTGTTCCTGCCTTTCGGTTTACCGGTGTCATTCCGCTGTTATGGCCGCGTTTGTCTCATTCCACGCCTGACACTCAGTTCCGGGTAGGCAGTTCGCTCCAAGCTGGACTGTATGCACGAACCCCCCGTTCAGTCCGACCGCTGCGCCTTATCCGGTAACTATCGTCTTGAGTCCAACCCGGAAAGACATGCAAAAGCACCACTGGCAGCAGCCACTGGTAATTGATTTAGAGGAGTTAGTCTTGAAGTCATGCGCCGGTTAAGGCTAAACTGAAAGGACAAGTTTTGGTGACTGCGCTCCTCCAAGCCAGTTACCTCGGTTCAAAGAGTTGGTAGCTCAGAGAACCTTCGAAAAACCGCCCTGCAAGGCGGTTTTTTCGTTTTCAGAGCAAGAGATTACGCGCAGACCAAAACGATCTCAAGAAGATCATCTTATTAAGGGGTCTGACGCTCAGTGGAACGAAAACTCACGTTAAGGGATTTTGGTCATGAGATTATCAAAAAGGATCTTCACCTAGATCCTTTTAAATTAAAAATGAAGTTTTAAATCAATCTAAAGTATATATGAGTAAACTTGGTCTGACAGTTACCAATGCTTAATCAGTGAGGCACCTATCTCAGCGATCTGTCTATTTCGTTCATCCATAGTTGCCTGACTCCCCGTCGTGTAGATAACTACGATACGGGAGGGCTTACCATCTGGCCCCAGTGCTGCAATGATACCGCGAGACCCACGCTCACCGGCTCCAGATTTATCAGCAATAAACCAGCCAGCCGGAAGGGCCGAGCGCAGAAGTGGTCCTGCAACTTTATCCGCCTCCATCCAGTCTATTAATTGTTGCCGGGAAGCTAGAGTAAGTAGTTCGCCAGTTAATAGTTTGCGCAACGTTGTTGCCATTGCTGCAGGCATCGTGGTGTCACGCTCGTCGTTTGGTATGGCTTCATTCAGCTCCGGTTCCCAACGATCAAGGCGAGTTACATGATCCCCCATGTTGTGCAAAAAAGCGGTTAGCTCCTTCGGTCCTCCGATCGTTGTCAGAAGTAAGTTGGCCGCAGTGTTATCACTCATGGTTATGGCAGCACTGCATAATTCTCTTACTGTCATGCCATCCGTAAGATGCTTTTCTGTGACTGGTGAGTACTCAACCAAGTCATTCTGAGAATAGTGTATGCGGCGACCGAGTTGCTCTTGCCCGGCGTCAACACGGGATAATACCGCGCCACATAGCAGAACTTTAAAAGTGCTCATCATTGGAAAACGTTCTTCGGGGCGAAAACTCTCAAGGATCTTACCGCTGTTGAGATCCAGTTCGATGTAACCCACTCGTGCACCCAACTGATCTTCAGCATCTTTTACTTTCACCAGCGTTTCTGGGTGAGCAAAAACAGGAAGGCAAAATGCCGCAAAAAAGGGAATAAGGGCGACACGGAAATGTTGAATACTCATACTCTTCCTTTTTCAATATTATTGAAGCATTTATCAGGGTTATTGTCTCATGAGCGGATACATATTTGAATGTATTTAGAAAAATAAACAAATAGGGGTTCCGCGCACATTTCCCCGAAAAGTGCCACCTGACGTGTCGACGCGGCCGCTAGCGATGACCCTGCTGATTGGTTCGCTGACCATTTCCGGGTGCGGGACGGCGTTACCAGAAACTCAGAAGGTTCGTCCAACCAAACCGACTCTGACGGCAGTTTACGAGAGAGATGATAGGGTCTGCTTCAGTAAGCCAGATGCTACACAATTAGGCTTGTACATATTGTCGTTAGAACGCGGCTACAATTAATACATAACCTTATGTATCATACACATACGATTTAGGTGACACTATAG
