# Supplementary material for: Generation of a reporter yellow fever virus for high throughput antiviral assays
Source: Antiviral Res. 2020 Nov;183:104939. doi: 10.1016/j.antiviral.2020.104939 (PMC7649875; doi:10.1016/j.antiviral.2020.104939)
Supplement: Multimedia component 3 [file mmc3.docx]

**Supplementary Sequence S2. Fragment containing HiBiT tag.** The fragment containing the HiBiT tag was synthesised. Primer binding sites used to amplify this region is underlined. Fragment containing the first 4 amino acids of NS1-HiBiT tag and GSSG linker is in bold letters.

CAGCTCCGCTGGAGGGTTCTTCACTTCGGTTGGGAAAGGAATTCATACGGTGTTTGGCTCTGCCTTTCAGGGGCTATTTGGCGGCTTGAACTGGATAACAAAGGTCATCATGGGGGCGGTACTTATATGGGTTGGCATCAACACAAGAAACATGACAATGTCCATGAGCATGATCTTGGTAGGAGTGATCATGATGTTTTTGTCTCTAGGAGTTGGGGCG**GACCAGGGTTGTGTGAGCGGCTGGCGGCTGTTCAAGAAGATTAGCgggagttctggc**GATCAAGGATGCGCCATCAACTTTGGCAAGAGAGAGCTCAAGTGCGGAGATGGTATCTTCATATTTAGAGACTCTGATGACTGGCTGAACAAGTACTCATACTATCCAGAAGATCCTGTGAAGCTTGCATCAATAGTGAAAGCCTCTTTTGAAGAAGGGAAGTGTGG
